# Serum concentrations of mood stabilizers are associated with memory, but not other cognitive domains in psychosis spectrum disorders; explorative analyses in a naturalistic setting

**DOI:** 10.1186/s40345-016-0067-z

**Published:** 2016-12-09

**Authors:** Nils Eiel Steen, Monica Aas, Carmen Simonsen, Ingrid Dieset, Martin Tesli, Mari Nerhus, Erlend Gardsjord, Ragni Mørch, Ingrid Agartz, Ingrid Melle, Anja Vaskinn, Olav Spigset, Ole A. Andreassen

**Affiliations:** 1 NORMENT, KG Jebsen Centre for Psychosis Research, Division of Mental Health and Addiction, Oslo University Hospital, and Institute of Clinical Medicine, University of Oslo, Oslo, Norway; 2 Drammen District Psychiatric Center, Clinic of Mental Health and Addiction, Vestre Viken Hospital Trust, 3004 Drammen, Norway; 3 Lovisenberg Diakonale Hospital, Oslo, Norway; 4 Department of Psychiatric Research, Diakonhjemmet Hospital, Oslo, Norway; 5 Department of Psychology, University of Oslo, Oslo, Norway; 6 Department of Clinical Pharmacology, St. Olav University Hospital, Trondheim, Norway; 7 Department of Laboratory Medicine, Children’s and Women’s Health, Norwegian University of Science and Technology, Trondheim, Norway

**Keywords:** Mood stabilizers, Cognition, Neuropsychological test, Psychosis, Bipolar disorder, Schizophrenia

## Abstract

**Background:**

Mood stabilizers like lithium and anticonvulsants are used in bipolar and related psychotic disorders. There is a lack of knowledge of the relationship of these medications and cognition in the psychosis spectrum. We studied the association between serum concentration of mood stabilizers and cognitive performance in a well-characterized sample of bipolar and schizophrenia spectrum disorders.

**Methods:**

Serum concentrations of valproate, lamotrigine, and lithium were analyzed for associations to performance on neuropsychological tests in six cognitive domains in individuals with bipolar disorder (*n* = 167) and in a combined sample of individuals with bipolar or schizophrenia spectrum disorders (*n* = 217). Linear regression with adjustments for gender, age, and symptom levels of depression, mania, and psychosis were applied for the association analyses.

**Results:**

There were negative associations between serum levels of valproate and short term delayed recall (bipolar: *p* = 0.043; combined: *p* = 0.044) and working memory (bipolar: *p* = 0.043). A positive association was suggested between serum level of lithium and working memory (bipolar: *p* = 0.039). There were no other significant relationships between serum levels of valproate, lamotrigine, or lithium and neuropsychological test performance in neither the bipolar disorder nor the combined group.

**Conclusions:**

Serum levels of mood stabilizers were unrelated to cognitive performance in most domains, indicating that higher dose does not lead to broader cognitive impairments in bipolar and related psychotic disorder patients. However, worsened memory with increasing levels of valproate suggests cautious dosing of anticonvulsants, while increasing lithium level seems to be associated with improved memory. The findings should be interpreted with caution due to the explorative, naturalistic design.

## Background

Cognitive impairment is well documented in bipolar and related psychosis spectrum disorders (Bourne et al. 2013; Vaskinn et al. 2014). Both decreased and superior cognitive abilities seem to be associated with contracting bipolar disorder (Bora 2015), while history of psychosis (Simonsen et al. 2011), duration of illness, mood episodes, and hospitalizations (Cardoso et al. 2015) have been associated with differences in cognitive performance after disease onset. Cognitive domains typically affected include memory, attention, processing speed, and executive functioning (Bourne et al. 2013), with the impairments having a substantial impact on social and occupational outcome (Baune and Malhi 2015). The need to address the cognitive aspect in treatment is obvious; however, research on cognitive implications of standard medication in bipolar and related disorders is sparse.

Lithium and the anticonvulsant agents valproate and lamotrigine are classical mood stabilizers used in the treatment of bipolar disorder. They are also used as adjunctives in schizophrenia in attempts to optimize treatment effect (Citrome 2009). Lithium has been found to negatively influence verbal memory and psychomotor speed; however, there are as well indications of brain protective effects, suggesting cognitive benefits. Valproate has been associated with impaired cognitive functioning in several domains, while studies of lamotrigine indicate less negative influences, even advantageous effects for certain domains such as memory and verbal fluency have been suggested (see Dias et al. 2012 for an overview of mood stabilizers and cognition).

One central factor for assessing cognitive performance during treatment with mood stabilizers is the actual level of drug exposure. This is shown in animal studies (Honarmand et al. 2014), human dementia studies (Leyhe et al. 2009), as well as in epilepsy samples. In psychiatric samples, there are some preliminary findings suggesting effects of serum level of lithium; both worsened verbal learning, memory, and executive function (Bora et al. 2007; Squire et al. 1980) and improved short term memory (Squire et al. 1980) associated with higher serum levels are suggested. However, generally, there is no solid evidence supporting that lithium enhances memory in any meaningful fashion. Relations between serum levels of valproate and cognitive performance have been studied in patients with epilepsy; these studies indicate worsened cognition or no association with cognitive performance with higher serum concentrations (Brouwer et al. 1992; Gallassi et al. 1990; Jakovljevic et al. 2008; Prevey et al. 1996; Trimble and Thompson 1984). For lamotrigine, there is a lack of data of the relationship between serum concentration and cognitive performance both in epilepsy and psychosis spectrum disorders.

In the present study, we investigated the relationships between serum concentrations of mood stabilizers and cognitive functioning in bipolar and related psychotic disorders. This is done in a large and well-characterized sample, enabling adjustments for several confounders. Using serum levels instead of dosage, the regular biases due to adherence issues and interindividual variations in pharmacokinetics are avoided. Moreover, blood sampling was performed the same morning as the neuropsychological battery, enabling an almost direct comparison. Due to the sparse literature, the analyses were basically explorative, although it was possible to make a few expectations. For valproate, we expected no associations or tentatively worsened cognitive performance with increasing serum levels. For lithium, we expected no associations or tentatively worsened verbal learning, memory, and executive function and improved short term memory with increasing serum levels. For lamotrigine, the analyses were explorative.

## Methods

### Participants

Patients were included through the ongoing thematically organized psychosis (TOP) study carried out by the University Hospitals of Oslo, Norway. The TOP study is a multicenter study focusing on neurobiological, genetic, psychologic, and environmental mechanisms behind psychosis spectrum disorders. Patients are recruited through referrals from in- and out-patient mental health clinics with a catchment area of basically the city of Oslo. General inclusion criteria for the TOP study are being registered in the psychiatric services of any one of the participating hospitals, age 18–65 years, meeting the diagnostic and statistical manual of mental disorders (DSM)-IV (American Psychiatric Association 2000) criteria for bipolar or schizophrenia spectrum disorders, and being willing and able to give written, informed consent to participation. General exclusion criteria are a history of moderate or severe head injury, serious somatic illness, neurological disorder, and mental retardation (see also Birkenaes et al. 2007 for a broader description).

Included in the current analyses were consecutively referred patients from 2003 to 2014 with a DSM-IV bipolar disorders diagnosis, in the following termed “bipolar disorder” (BD, *N* = 167), or a schizophrenia and other psychotic disorders diagnosis, in the following termed “schizophrenia” (SCZ, *N* = 50), with neuropsychological testing and concurrent measurements of serum levels of lamotrigine or valproate recorded as primary anticonvulsant drug, or concurrent measurements of serum levels of lithium. Selection of anticonvulsants was based on the prescription frequency in the sample. Table 1 shows the sample characteristics.

**Table 1:** Demographic data, diagnosis, and psychopharmacological treatment

	Median	IQR
Age (years)	33.0	17.0
IDS, total score	15.0	18.0
YMRS, total score	2.0	8.0
SCI-PANSS, total score	46.0	17.0

	N	%
Total number of participants	217	100
Number of males	89	41.0
Diagnosis		
Schizophrenia and other psychotic disorders^a^	50	23.0
Schizophrenia	21	9.7
Schizophreniform disorder	2	0.9
Schizoaffective disorder	17	7.8
Delusional disorder	1	0.5
Brief psychotic disorder	1	0.5
Psychotic disorder NOS	8	3.7
Bipolar disorders^a^	167	77.0
Bipolar I disorder	117	53.9
Bipolar II disorder	42	19.4
Bipolar disorder NOS	8	3.7
Main anticonvulsant drug^b^		
Lamotrigine	109	50.2
Valproate	62	28.6
Lithium^c^	58	26.7
More than one mood stabilizer^d^	17	7.8
Other psychotropic drugs		
Use of other anticonvulsant drugs	11	5.1
Use of antipsychotics	128	59.0
Use of antidepressants	86	39.6
Use of sedatives^e^	35	16.1

*BD* bipolar disorder, *IDS* inventory of depressive symptomatology, *IQR* interquartile range, *N* number, *NOS* not otherwise specified, *SCI-PANSS* structured clinical interview for the positive and negative syndrome scale, *SCZ* schizophrenia and other psychotic disorders, *YMRS* Young mania rating scale

^a^DSM-IV (American Psychiatric Association 2000)

^b^Duration of treatment (months, median [IQR]): 4.0 (13.6); participants with BD: *N* = 82 (lamotrigine), *N* = 43 (valproate)

^c^Duration of treatment (months, median [IQR]): 8.0 (64.3); participants with BD: *N* = 52

^d^Of these *N* = 5 used lamotrigine + valproate, *N* = 4 used valproate + lithium, and *N* = 8 used lamotrigine + lithium

^e^Defined as hypnotics, anxiolytics, or sedatives

### Clinical assessments

Inclusion and diagnostic interviews were done by trained medical doctors and psychologists using The Structured Clinical Interview for DSM-IV Axis I Disorders, SCID 1 (First et al. 1995). Inter-rater reliability was good, with an overall kappa score of 0.77 (95% C.I 0.60–0.94). The inventory of depressive symptomatology-clinician rated (IDS-C) (Rush et al. 1996), Young mania rating scale (YMRS) (Young et al. 1978), the structured clinical interview for the positive and negative syndrome scale (SCI-PANSS) (Kay et al. 1987), and global assessment of functioning, symptom scale (GAF-S) (Pedersen et al. 2007) were used for symptom assessments (Table 1).

We recorded dosage of antipsychotics and antidepressants for the purpose of statistical adjustments. To combine different drugs within these categories, we calculated the ratio of the reported dose to the defined daily dose of the drug (DDD) (ATC/DDD Index 2016, WHO), using the sum of the ratios if a subject used more than one drug from a category. If a subject did not use any medication within a category, the value was set to zero. Information about pharmacological treatment was obtained from interviews and medical records. See also previous papers by Steen et al. (2011, 2015).

### Neuropsychological assessments

Within 2 weeks after clinical assessments, a 3-h neuropsychological test battery was administered in a fixed order with 2 refreshment breaks (Simonsen et al. 2011). Clinical psychologists with training in standardized neuropsychological testing administered the tests, supervised by a professor in neuropsychology, who also was in charge of the quality control. Test scoring was initially calibrated across investigators on the verbal tests from the Wechsler abbreviated scale of intelligence (WASI) (Wechsler 2007) in order to assure a common scoring technique. Six neuropsychological domains were selected for analyses. *Verbal learning* was measured by the California verbal learning test (CVLT) subscore Total A1–5 (Delis et al. 2004). *Verbal memory* was divided into “immediate recall”, “short term delayed recall”, and “long term delayed recall”. Immediate recall was assessed using the logical memory test subscore immediate recall from the Wechsler memory scale (Wechsler et al. 2008). Short term delayed recall was assessed using the CVLT subscore short delay free recall. Long term delayed recall was assessed using the CVLT subscore long delay free recall, and the logical memory test subscore delayed recall. *Attention* was assessed using the digit span test forward from the Wechsler adult intelligence scale (WAIS) (Wechsler 2003). *Working memory* was assessed using the digit span test backward from WAIS. *Executive functioning* was assessed using tests from the Delis–Kaplan executive function scale (D-KEFS), more specifically the verbal fluency test, with subscore measures of phonetic fluency and semantic fluency, and the interference control test subscore color-word inhibition and color-word inhibition/switching (Delis et al. 2005). *Processing speed* was assessed using the digit symbol coding test from WAIS, and the interference control test subscore color-word color-naming from D-KEFS. Table 2 shows the neuropsychological scores. Raw-scores were used across cognitive domains.

**Table 2:** Serum concentration of mood stabilizers, and neuropsychological test scores

	BD		SCZ	
	Median	IQR	Median	IQR
Serum concentrations of mood stabilizers^a^				
Lamotrigine (µmol/L)	10.4	13.1	8.4	10.4
Valproate (µmol/L)	304	336	386	428
Lithium (mmol/L)	0.58	0.27	0.60	0.46
Neuropsychological test scores				
Verbal learning				
CVLT Total A1-5	56.0	15.0	48.0	15.3
Verbal memory—immediate recall				
Logical memory test immediate recall	24.0	10.0	20.5	10.5
Verbal memory—short term delayed recall				
CVLT short delay free recall	13.0	5.0	11.0	5.5
Verbal memory—long term delayed recall				
CVLT long delay free recall	13.0	4.0	12.0	5.3
Logical memory test delayed recall	22.0	11.0	16.0	11.5
Attention				
Digit span test forwards	6.0	2.0	6.0	2.0
Working memory				
Digit span test backwards	4.0	1.0	4.0	2.0
Executive function				
Verbal fluency test letter^b^	43.0	18.3	33.0	25.0
Verbal fluency test category^c^	44.0	12.0	35.5	16.3
CW 3 inhibition	56.0	18.3	64.0	28.3
CW 4 inhibition/switching	60.0	18.0	65.0	29.5
Processing/psychomotor speed				
Digit symbol coding test	67.0	21.0	53.0	24.3
CW Stroop (color naming)	31.0	9.0	38.0	13.0

*SCZ* schizophrenia and other psychotic disorders, *BD* bipolar disorder, *IQR* interquartile range, *CVLT* California verbal learning test, *CW* color-word

^a^There were no significant differences in serum concentrations of mood stabilizers between BD and SCZ

^b^Scores were missing for 6% of subjects in the SCZ group

^c^Scores were missing for 8% of subjects in the SCZ group

### Serum concentration measurements of anticonvulsant drugs and lithium

Blood was drawn from the antecubital vein in the morning the same day as the neuropsychological testing took place. The patients met fasting for blood sampling without taking their morning medication. Thus, blood for serum levels measurements were collected on average 12 or 24 h after last dose according to standard laboratory requirements. After blood sampling, the patients took their morning medication, had a light breakfast and a brief physical examination. Then the neuropsychological testing was done with a standard sequence of tests starting on average 1 h after blood sampling. The laboratories and breakfast room were located close to each other in the same building. Serum concentrations of lamotrigine were analyzed with a liquid chromatography–mass spectrometry (LC–MS) method developed in our laboratory and described in detail elsewhere (Reimers et al. 2005). Serum concentrations of valproate and lithium were analyzed by means of commercially available kits using a Cobas Integra 400 plus system (Roche Diagnostics, Rotkreutz, Switzerland). Table 2 shows the serum concentrations.

### Ethics

The study was carried out in accordance with the Declaration of Helsinki. After complete description of the study to the subjects, written informed consent was obtained. The Regional Ethics Committee South-East and The Norwegian Data Inspectorate approved the study. The biobank was approved by The Norwegian Directorate of Health.

### Statistical analysis

Neuropsychological test scores from the six domains were tested for associations with serum concentrations of lamotrigine, valproate, and lithium using linear regressions. This was done for all three compounds in the BD sample and in the combined BD and SCZ sample, respectively. Neuropsychological test scores were set as dependent variables. For the BD sample, gender, age, serum concentration of the medication, and total scores of IDS-C and YMRS (lamotrigine and lithium), or GAF-S (valproate, due to smaller sample) were set as independent variables. For the combined BD and SCZ sample, gender, age, IDS-C, YMRS, SCI-PANSS, and serum concentration were set as independent variables. The final basic regression models were decided with a backwards elimination procedure using a significance threshold of ≥0.1 for elimination of excessive independent variables. The dependent variables were transformed using logarithmic, squared, or reciprocal transformation when required. Based on our previous findings of associations between serum levels of antidepressants and antipsychotics and cognitive performance (Steen et al. 2015, 2016), we also did extended statistical analyses in the combined sample by including dosage of antidepressants and antipsychotics, respectively, as independent variables. For descriptive purposes, we did association analyses between the cognitive measures, and between daily doses and serum levels of the mood stabilizers, using Spearman’s rho. For testing between group differences of serum concentrations, we used Mann–Whitney U test.

## Results

Cognitive test scores correlated significantly across the cognitive tests in the total sample (Spearman’s rho = 0.15–0.90 [absolute values], *p* = <0.001 to 0.031). Daily drug doses and serum concentrations correlated significantly for each drug (lamotrigine: Spearman’s rho = 0.77, *p* < 0.001; valproate: Spearman’s rho = 0.54, *p* < 0.001; lithium: Spearman’s rho = 0.34, *p* = 0.009).

### Associations between serum concentrations of anticonvulsants and cognitive performance

#### BD sample

##### Significant associations in the BD sample

**Fig. 1:**
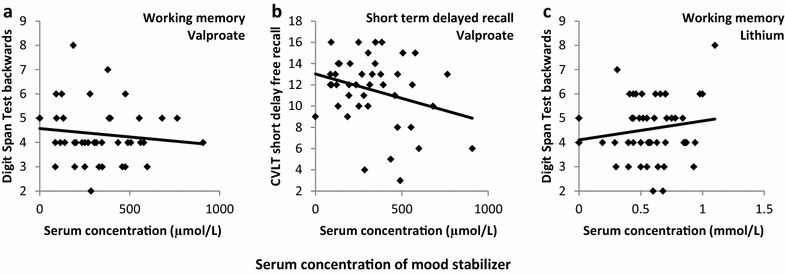
Scatter plots of the serum concentrations (*x-axis*) of valproate (µmol/L) (**a**, **b**) and lithium (mmol/L) (**c**) and neuropsychological test scores (*y-axis*) of digit span test backwards (**a**, **c**), and California verbal learning test (CVLT) short delay free recall (**b**) in the bipolar disorder sample

Negative associations were suggested between serum concentration of valproate and working memory and short term delayed recall. This was shown by significant main effects in the regressions for valproate (digit span test backward [*β* = −0.28, *p* = 0.043]; CVLT subscore short delay free recall [*β* = −0.30, *p* = 0.043]) (Fig. 1a, b).

#### Combined sample

##### Significant and trend-level significant associations in the combined sample

Negative associations were suggested between serum concentration of valproate and short term delayed recall, long term delayed recall, and working memory, and between serum concentrations of lamotrigine and working memory. This was shown by a significant main effect in a regression for valproate (CVLT subscore short delay free recall [standardized coefficient [*β*] = −0.26, *p* = 0.044]), and trend-level significant main effects in regressions for valproate (CVLT subscore long delay free recall [*β* = −0.24, *p* = 0.055, criteria of normally distributed residuals violated]; digit span test backward [*β* = −0.24, *p* = 0.058]) and lamotrigine (digit span test backward [*β* = −0.18, *p* = 0.058, before backward elimination: *β* = −0.19, *p* = 0.046]).

There were no other associations indicated between serum concentrations of lamotrigine or valproate and neuropsychological test scores in the BD or the combined sample in any of the tested cognitive domains, as shown by no significant main effects of the serum concentrations of lamotrigine or valproate in the linear regressions.

### Associations between serum concentration of lithium and cognitive performance

#### BD sample

##### Significant and trend-level significant associations in the BD sample

Data were suggestive of positive associations between serum concentration of lithium and working memory and attention as shown by the main effects in the regressions for lithium (digit span test backward [*β* = 0.29, *p* = 0.039, Fig. 1c]; digit span test forward [*β* = 0.24, *p* = 0.087]).

#### Combined sample

##### Trend-level significant associations in the combined sample

Data were slightly suggestive of positive associations between serum concentration of lithium and attention and working memory as shown by the main effects in the regressions for lithium (digit span test forward [non-significant after backwards elimination procedure, before backward elimination: *β* = 0.27, *p* = 0.056]; digit span test backward [*β* = 0.23, *p* = 0.081, non-significant after removing an observation with Cook’s distance = 0.40 and centered leverage value = 0.20]).

There were no other associations indicated between serum concentrations of lithium and neuropsychological test scores in the BD or the combined sample in any of the tested cognitive domains, as shown by no significant main effects of the serum concentrations of lithium in the linear regressions.

## Discussion

The main finding in the present study is no association between serum levels of lamotrigine, valproate, or lithium and performance on most neuropsychological tests from a broad test battery in individuals with bipolar and schizophrenia spectrum disorders. Significant associations suggested worsened short term delayed recall and working memory with increasing valproate concentration, and better working memory with increasing lithium concentration in BD. Findings in the combined BD and SCZ sample corresponded to those in BD for valproate.

Except from potentially negative effects of anticonvulsants on memory, the present findings indicate that the risk of broader cognitive side effects due to increasing the dosage of lamotrigine, valproate, or lithium is small. Correspondingly, there neither seems to be clinically significant cognitive benefits by adjusting the dosage. This is in line with previous studies showing that neuropsychological test performance is mainly not associated with the drug serum concentrations of valproate and lithium (Bora et al. 2007; Prevey et al. 1996). However, there are a few indications in the scientific literature of effects of the level of exposure, and the present data suggesting negative associations between memory and serum level of valproate are in line with such reports (Jakovljevic et al. 2008; Prevey et al. 1996). Moreover, although negative associations between serum lithium and memory also have been reported (Bora et al. 2007), the present positive association between serum lithium and working memory is in line with a previously indicated association between serum lithium and short term memory in correlation analyses in the study by Squire et al. (1980). As discussed by Cowan (2008), working memory and short term memory are regarded as closely associated abilities.

The previously suggested worsened memory and executive function with increasing lithium exposure (Bora et al. 2007; Squire et al. 1980) were not replicated in the present study. This may depend on methodological differences. In the study by Bora et al. (2007), the sample consisted of euthymic bipolar I disorder patients, and with an unexplained criterion of minimum 2 manic episodes to be included. Moreover, there was no correction of significance level for multiple testing, and the *p* value was >0.035 for the reported correlation with memory. Thus, in addition to an unusual inclusion criterion, the reported negative association seems prone to type 1 error given the number of analyses. In the present study, we used a broader clinical sample, and with diagnostics following the DSM-IV criteria. In this way, the results seem more easily generalizable to everyday clinical practice. Similarly, in the cross-over study by Squire et al. (1980), the sample was small (*N* = 16), and most of the participants had a diagnosis of alcohol abuse or dependency. These factors are in contrast to the present study with a several-fold larger subsample using lithium, enabling adjustments for important variables and reducing the risk of spurious associations, and without a similar impact from substance abuse and dependency. Importantly, alcohol dependency has been associated with cognitive decrements (Bernardin et al. 2014).

The processing of data was performed without taking into account reference serum values of the drugs. This was done as there is no a priori reason to believe that the biological effects on cognitive performance are confined to the therapeutic reference intervals for treating affective symptoms. Moreover, we wanted to gain knowledge of the potential association between serum levels of mood stabilizers and cognitive performance irrespective of the treatment effect. In line with this, we adjusted for symptom load in the statistical analyses. However, it should be noted that the median values of the serum concentrations (Table 2) of valproate in the BD group and of lamotrigine in both the BD and the SCZ groups were below the recommended reference intervals (Hiemke et al. 2011). Although Fig. 1 indicates a fairly good spread of data, low serum concentrations in these groups might have limited the ability to uncover significant associations, and possibly also the relevance to a broader population receiving mood stabilizers.

The strength of the study includes the use of serum levels as independent variables. This helps prevent biases at the level of the neuropsychological test administrator, and eliminates uncertainties of medication adherence and differences in pharmacokinetics. Moreover, by blood sampling being performed the same morning as the neuropsychological testing, an accurate measure of the relevant drug level was obtained (Steen et al. 2015). The neuropsychological testers were calibrated and supervised, assuring the quality of the dependent variables. Moreover, the sample is large compared to previous studies, enabling adjustments for several confounders. Due to the last couple of decades’ generation of knowledge of the cognitive impairments in psychosis spectrum disorders (Bourne et al. 2013; Kahn and Keefe 2013), a detailed analysis of the effects of the commonly used psychotropic drugs is pertinent. We have previously shown relationships between serum levels of antidepressants and antipsychotics and cognitive performance (Steen et al. 2015, 2016), and the present findings complement the cognitive aspects of standard medication in bipolar and related disorders.

Some limitations have to be considered. There are few previous studies on the topic, making the analyses largely explorative and the indicated associations preliminary. Especially, there are minimal former data of psychiatric samples. Due to the cognitive impairments in these patients groups, we did not adjust the significance level, despite the number of tests, to reduce the risk of overlooking adverse cognitive effects (type II errors). However, with the open naturalistic design, non-randomized treatment and lack of control group, there is a possibility of missing associations, as the study is vulnerable to confounders, including use of co-medications. However, we were able to adjust for several important variables due to the considerable sample size, including use of antidepressants and antipsychotics. A significant impact from sedatives is unlikely, as only 16.1% of the participants were registered with such medication, of which about half were short-acting hypnotics taken in the evening. Moreover, the study did find negative associations between cognition and drug-levels, and the usual concern of naturalistic studies of higher drug doses used for increased severity of condition (Bolstad et al. 2011; for a discussion, see Ho et al. 2007) would rather strengthen negative associations. In addition to specific analyses of the BD sample, we analyzed the larger combined sample with the potential of discovering weaker associations; however, there were fewer significant findings in the combined sample, possibly due to larger heterogeneity. Heterogeneity might also have influenced the results in the BD sample, which included the complete DSM-IV bipolar disorders category. However, given the current sample size, it is unlikely that clinical meaningful associations would not have been detected. We did not perform specific analyses of SCZ due to the smaller sample size preventing appropriate statistical adjustments within each drug group. Moreover, it should be noted that lack of analyses of subjective cognitive complaints, might limit the generalizability of the findings to daily life. Lastly, the rather young age of the patients in the present sample should be noted. We included age as an independent variable in the analyses; however, specific analyses of older age groups in a larger sample would have been preferable based on previous suggested effects (Forester et al. 2009).

In the present study, we investigated the serum levels of commonly used mood stabilizers in relation to cognitive performance in six cognitive domains in a well-characterized sample of BD and SCZ. The main finding was no association between serum level of lamotrigine, valproate, or lithium and cognitive performance in most domains. A negative association for valproate and memory was indicated, and deserves further investigation in larger samples. The data were suggestive of a positive association between lithium level and working memory; however, the finding was too weak to indicate significant clinical implications. The present study of serum levels of mood stabilizers and cognition is one of the first studies in a considerable large sample of psychosis spectrum patients, and although in need of replication, gives reason to believe that lamotrigine, valproate, and lithium can be administered across the dosage ranges without broad cognitive adverse effects. However, higher doses of anticonvulsants might negatively affect memory.

## Abbreviations

BD: bipolar disorders
CVLT: California verbal learning test
DDD: defined daily dose
DSM: diagnostic and statistical manual of mental disorders
D-KEFS: Delis–Kaplan executive function scale
GAF-S: global assessment of functioning, symptom scale
IDS-C: inventory of depressive symptomatology-clinician rated
LC–MS: liquid chromatography–mass spectrometry
SCI-PANSS: structured clinical interview for the positive and negative syndrome scale
TOP study: thematically organized psychosis study
SCZ: schizophrenia and other psychotic disorders
WAIS: Wechsler adult intelligence scale
WASI: Wechsler abbreviated scale of intelligence
YMRS: Young mania rating scale
